# Dissociative Amnesia with Regressive Features in a Patient with Multiple Sclerosis: A Case Report

**DOI:** 10.1192/j.eurpsy.2026.11082

**Published:** 2026-07-31

**Authors:** Z. L. Onat, S. Kükürt

**Affiliations:** Psychiatry, Bezmialem Vakıf University, Istanbul, Türkiye

## Abstract

**Introduction:**

Multiple sclerosis (MS) is a chronic neuroinflammatory disorder frequently associated with psychiatric comorbidities such as depression, anxiety, bipolar disorder, and psychosis. However, dissociative disorders in MS are rarely reported and remain underexplored. Dissociative amnesia accompanied by regressive behaviors is likely influenced by both the psychological burden of MS and neurocognitive vulnerabilities.

**Objectives:**

To present a rare case of MS with dissociative amnesia and prominent regressive features, and to discuss its clinical relevance in the context of psychiatric comorbidities of MS.

**Methods:**

We report the clinical course, psychiatric evaluation, neuroimaging findings, and treatment of a 24-year-old woman with MS, diagnosed at age 16 and without prior psychiatric history. Symptom severity was measured with the Dissociative Symptoms Scale-Brief (DSS-B).

**Results:**

The patient developed acute transient loss of consciousness, and retrograde amnesia. Neuropsychological findings included prosopagnosia, agnosia, anomia, puerile speech, la belle indifference, and childlike behaviors. The DSS-B score was 30, indicating a high level of dissociative symptoms and consistent with clinically observed amnesia and regressive behaviors. MRI revealed chronic periventricular plaques and a new hyperintense lesion at C4; however, the location of this lesion does not account for the acute dissociative and regressive symptoms observed. Psychosocial stressors included ongoing divorce proceedings and financial strain related to her spouse’s gambling. She was prescribed fluoxetine (20 mg/day) and trifluoperazine (1 mg/day). Two weeks later, she was re-evaluated, and her symptoms persisted with no noticeable improvement. This duration is longer than typically observed in dissociative amnesia. She subsequently discontinued treatment.

**Conclusions:**

This case demonstrates the rare occurrence of dissociative amnesia with regressive features in MS, highlighting the potential contribution of both neurocognitive and psychosocial factors. The prolonged duration of symptoms emphasizes the atypical nature of this presentation and suggests the need for careful multidisciplinary assessment.

**Disclosure of Interest:**

None Declared

